# Cystic Thymoma Masquerading as Simple Pericardial Cyst

**DOI:** 10.1016/j.atssr.2024.07.016

**Published:** 2024-07-31

**Authors:** Jennifer M. Nishimura, Camille Yongue, Fang Zhou, Stephanie H. Chang

**Affiliations:** 1 Division of Cardiothoracic Surgery, Department of Surgery, University of New Mexico, Albuquerque, New Mexico; 2 Division of Thoracic Surgery, Department of Cardiothoracic Surgery, New York University Langone Health, New York, New York; 3 Department of Pathology, New York University Langone Health, New York, New York

## Abstract

Cystic degeneration of thymoma can occur, although rarely to the extent that the lesion appears entirely cystic. We present a case of a 26-year-old man with a large anterior mediastinal cyst that was resected with histopathologic examination revealing a cystic thymoma.

Thymomas are epithelial tumors that originate in the thymus. Although rare overall, they are the most common primary neoplasm in the anterior mediastinum.^1^ Focal cystic degeneration can often occur, but rarely to the extent that the mass becomes mostly or entirely cystic.^2^ We present a case of a 26-year-old man with a large right anterior pleural cyst, which resembled a pericardial cyst, that was resected. Histopathologic examination revealed a cystic thymoma.

A 26-year-old man presented with a large cystic mass in the right pleural space, which was found incidentally on chest radiography. He was asymptomatic, with no significant past medical history. On chest computed tomography (CT), it was thin walled and well circumscribed and measured 12.6 × 10.1 cm (Figure 1), consistent with a pericardial cyst. Tumor markers were within the reference range. Results of pulmonary function tests suggested a restrictive pattern of lung disease of extrapulmonary cause. Given its large size, resultant compressive atelectasis from its mass effect, and potential for enlargement, the patient decided to undergo surgical resection of the lesion.

**Figure 1:**
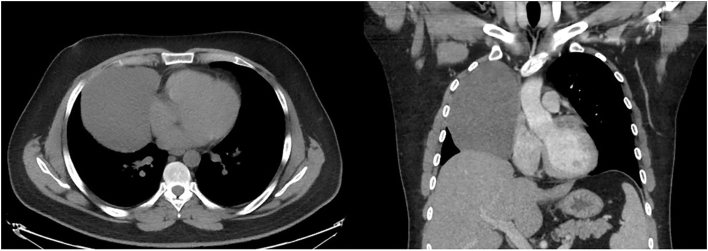
Chest computed tomography demonstrating a cystic mass in the anterior mediastinum.

A right robot-assisted thoracoscopic approach was taken with the patient in the left lateral decubitus position. The large cystic mass was located anterior to the phrenic nerve, entirely within the right side of the chest, with no other abnormalities. It had an appearance of a simple cyst, fluid filled and thin walled. To aid in dissection, the cyst contents were evacuated with a syringe, removing 900 mL of thin, serous fluid. The cyst was resected. A small stalk connected the cyst to the anterior pericardium, so the stalk was divided and removed with no residual specimen on the pericardium. The pericardium was left intact. He was discharged home the same day, with a normal postoperative course.

Final diagnosis after histopathologic examination was cystic thymoma (World Health Organization histologic classification, type B1), modified Masaoka stage I (Figure 2). The epithelial cells in the thymoma were positive for cytokeratin AE1/AE3 and p40 on immunostaining (Figure 3). In addition to the thymoma, the wall of the cyst contained residual nontumor thymic tissue with lymphoid hyperplasia and reactive cystic changes. There was attenuated and denuded epithelial lining in the cystic spaces. Cytologic assessment of the cystic fluid obtained intraoperatively revealed macrophages without lymphocytes or epithelial cells.

**Figure 2:**
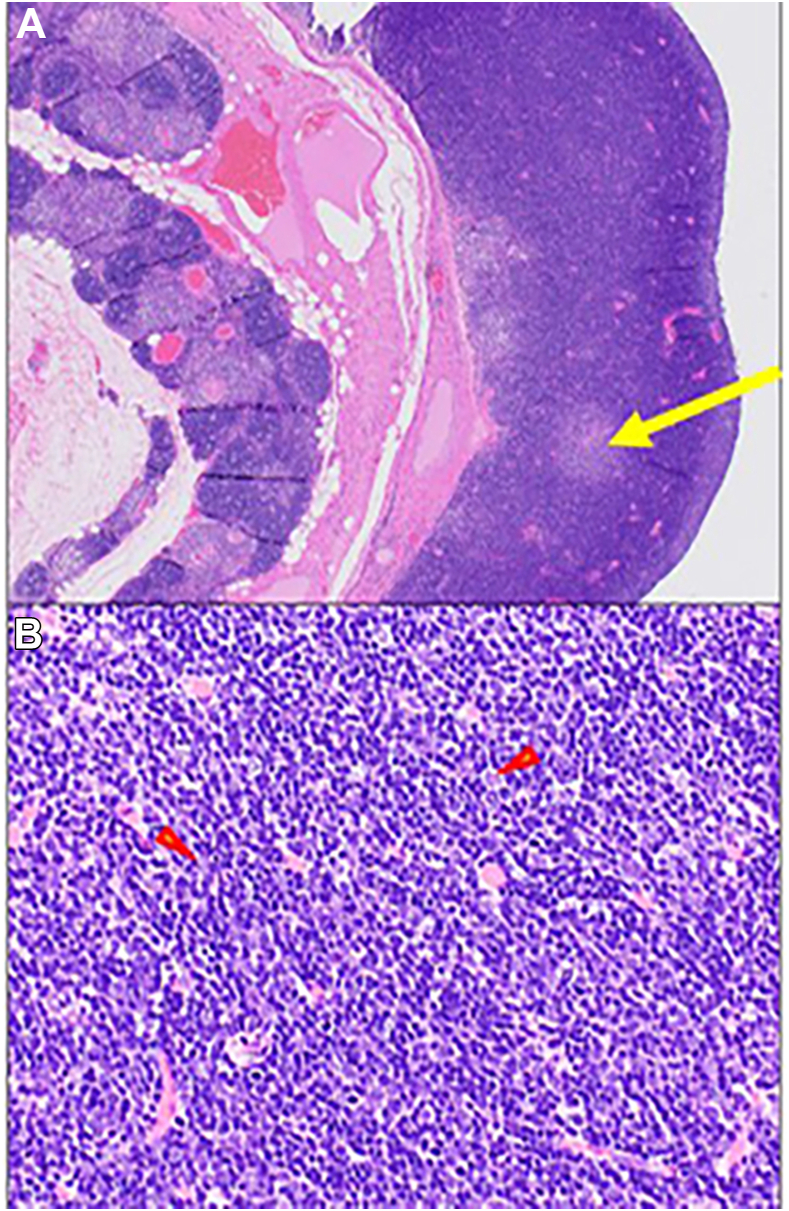
Type B1 thymoma resembles normal thymus but shows solid growth. (A) The thymoma is seen on the inner surface of the cyst (right side of figure); benign thymic tissue is seen on the outer aspect of the cyst (left side of figure). A medullary island is seen in the thymoma (arrow; hematoxylin and eosin stain, magnification ×20). (B) Similar to normal thymus, a B1 thymoma contains dispersed epithelial cells (arrowheads) in a dense background of thymic T cells (hematoxylin and eosin stain, magnification ×200).

**Figure 3:**
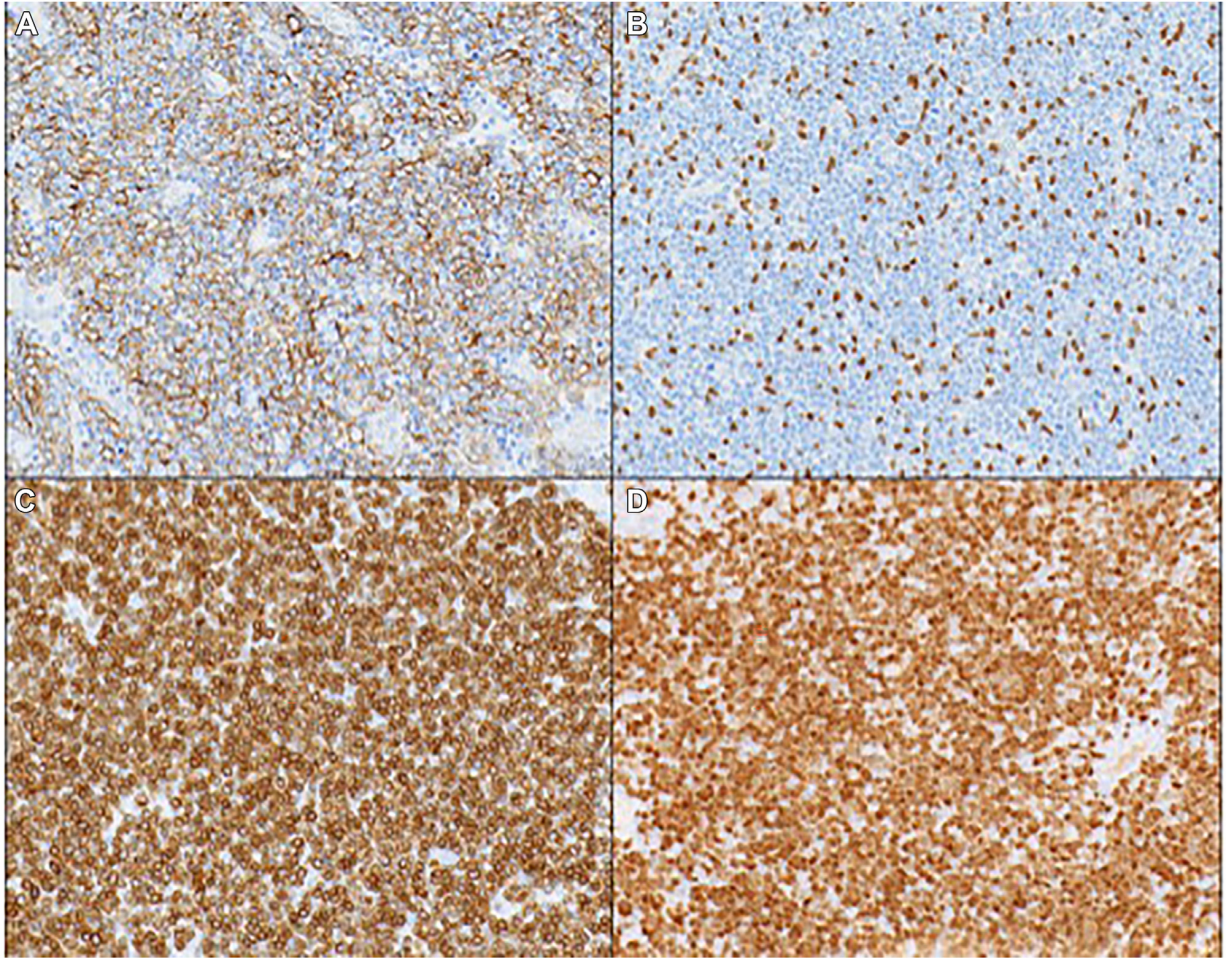
Immunostains (magnification ×200). The epithelial cells in the thymoma are positive for cytokeratin AE1/AE3 (A), which shows a delicate lacelike network pattern, and p40 (B). The thymic lymphocytes are positive for CD3 (C) and TdT (D).

Chest CT performed 2 months later showed a small amount of ill-defined soft tissue in the anterior mediastinum probably representing residual thymic tissue, with no evidence of any recurrent or distant disease. Six months after initial resection, he returned to the operating room for robot-assisted thoracoscopic completion thymectomy through a right-sided approach. On entry, the pleura was examined, and no implants were visualized. Completion thymectomy was performed, followed by parietal pleural biopsy to evaluate for microscopic metastatic disease. He was discharged home on the day of the operation. Final pathologic examination revealed benign thymic tissue, negative for neoplasm, with no evidence of metastatic disease in the pleural biopsy specimens. He has continued with serial imaging surveillance; 3.5 years after resection, there is no evidence of recurrent disease.

## Comment

The incidence of thymoma rises after the age of 40 years, peaks in the seventh decade of life, and is reported to be exceedingly uncommon in children and young adults.^1^ However, this trend may be different for cystic thymomas, which are increased in younger patients.^2^^,^^3^ When degeneration of thymoma occurs to the extent that the mass becomes entirely cystic, the ability to distinguish cystic thymomas from congenital or other benign cysts can be difficult, especially in young patients. CT imaging can often provide an accurate and definitive diagnosis of thymoma, especially in older patients, because of fatty involution of the thymus allowing easier identification of thymomas. The presence of mural soft tissue nodules of variable size and number is a characteristic feature of cystic thymoma.^4^ However, in the younger patient and in cases of thymoma with a nearly complete cystic morphology, CT imaging of cystic thymomas may be suggestive of a nonneoplastic process, such as a thymic or pericardial cyst. In this particular case, the patient had a simple pleura-based lesion that appeared consistent with a pericardial cyst. However, even in these situations, magnetic resonance imaging is indicated as it provides greater diagnostic precision, given its ability to better detect the presence of solid components or to reveal fibrous septa and nodularity, suggesting cystic degeneration or a cystic component of a solid neoplasm.^4^^,^^5^ In our patient, preoperative chest magnetic resonance imaging could have potentially helped distinguish between a pericardial cyst and a cystic thymoma, which would have led to different intraoperative management with increased caution during aspiration of the fluid and concurrent thymectomy.

It is important to distinguish cystic thymomas from congenital or other nonneoplastic cysts because management differs for these conditions. Current guidelines recommend total thymectomy for thymomas, whereas benign lesions can be observed or undergo cyst resection if indicated. Even after complete resection, thymomas tend to recur and can recur late, requiring long-term follow-up.^6^ If recurrence is identified, surgical resection can be effective and safe.^7^ Nonneoplastic cysts do not generally require imaging surveillance.

In this case, a 26-year-old man presented with a large cystic mass in the right pleural space, consistent with the diagnosis of pericardial cyst, which was resected. After histopathologic examination revealed the mass to be a cystic thymoma, the case was presented in multidisciplinary tumor board with consensus to perform a completion thymectomy. Concurrent pleurectomy was discussed, given concern that cystic fluid entered the pleural space during the procedure, but was not performed, given the lack of convincing evidence to support an indication for it. There is a paucity of data examining the clinical significance of cyst fluid entering the pleural space, and there was no evidence of pleural metastases on imaging or intraoperatively during completion thymectomy. This patient will continue to receive long-term surveillance for recurrence, including CT of the chest per current guidelines and standard of care. In conclusion, although thymoma is more commonly reported in patients 40 years of age or older, cystic thymoma should be considered in the differential when younger patients present with a cystic mass in the pleural space.
